# LMTK2-mediated Phosphorylation Regulates CFTR Endocytosis in Human Airway Epithelial Cells*

**DOI:** 10.1074/jbc.M114.563742

**Published:** 2014-04-11

**Authors:** Simão Luz, Kristine M. Cihil, David L. Brautigan, Margarida D. Amaral, Carlos M. Farinha, Agnieszka Swiatecka-Urban

**Affiliations:** From the ‡ Centre for Biodiversity, Functional and Integrative Genomics, University of Lisboa, 1749-016 Lisboa, Portugal,; the § Department of Nephrology, Children's Hospital of Pittsburgh, Pittsburgh, Pennsylvania 15201,; the ¶ Center for Cell Signaling and Department of Microbiology, Immunology, and Cancer Biology, University of Virginia School of Medicine, Charlottesville, Virginia 22908, and; the ‖ Department of Cell Biology, University of Pittsburgh School of Medicine, Pittsburgh, Pennsylvania 15261

**Keywords:** CFTR, Chloride Transport, Cystic Fibrosis, Endocytosis, Epithelial Cell, Protein Phosphorylation

## Abstract

Cystic fibrosis transmembrane conductance regulator (CFTR) is a Cl^−^-selective ion channel expressed in fluid-transporting epithelia. Lemur tyrosine kinase 2 (LMTK2) is a transmembrane protein with serine and threonine but not tyrosine kinase activity. Previous work identified CFTR as an *in vitro* substrate of LMTK2, suggesting a functional link. Here we demonstrate that LMTK2 co-immunoprecipitates with CFTR and phosphorylates CFTR-Ser^737^ in human airway epithelial cells. LMTK2 knockdown or expression of inactive LMTK2 kinase domain increases cell surface density of CFTR by attenuating its endocytosis in human airway epithelial cells. Moreover, LMTK2 knockdown increases Cl^−^ secretion mediated by the wild-type and rescued ΔF508-CFTR. Compared with the wild-type CFTR, the phosphorylation-deficient mutant CFTR-S737A shows increased cell surface density and decreased endocytosis. These results demonstrate a novel mechanism of the phospho-dependent inhibitory effect of CFTR-Ser^737^ mediated by LMTK2 via endocytosis and inhibition of the cell surface density of CFTR Cl^−^ channels. These data indicate that targeting LMTK2 may increase the cell surface density of CFTR Cl^−^ channels and improve stability of pharmacologically rescued ΔF508-CFTR in patients with cystic fibrosis.

## Teaser

**Background:** The CFTR-Ser^737^ site has a phospho-dependent inhibitory effect on Cl^−^ secretion.

**Results:** LMTK2 phosphorylation of CFTR-Ser^737^ facilitates endocytosis, reduces cell surface density of CFTR, and inhibits Cl^−^ secretion.

**Conclusion:** Targeting LMTK2 regulates the cell surface density of CFTR Cl^−^ channels.

**Significance:** Targeting LMTK2 in CF patients may stabilize ΔF508-CFTR pharmacologically rescued to the cell surface.

## Introduction

The cystic fibrosis transmembrane conductance regulator (CFTR),^3^ a member of the ATP binding cassette (ABC) transporter superfamily functions as a cAMP-activated Cl^−^-selective ion channel in various fluid-transporting epithelia (1–3). CFTR is present in many tissues including the airway where it plays a critical role in regulating mucociliary clearance by maintaining the homeostasis of the airway surface liquid (4, 5). CFTR-mediated Cl^−^ secretion across polarized epithelial cells is regulated by adjusting activity and density of the CFTR Cl^−^ channels at the cell surface (6–10). The cell surface abundance of CFTR depends on its biosynthetic processing and post-maturational trafficking, a process of endocytic uptake followed either by recycling to the plasma membrane or trafficking for lysosomal degradation (reviewed in Ref. 11). Despite inefficient biosynthetic processing, CFTR abundance at the cell surface is maintained after rapid endocytosis via clathrin-dependent pathway because CFTR is efficiently recycled (12–15). Deletion of Phe-508 (ΔF508) resulting from the most common CFTR gene mutation in patients with cystic fibrosis (CF) leads to a biosynthetic processing defect of the CFTR protein (11). Pharmacological correction of the processing defect has been highly anticipated as a disease modifying therapy because rescue of the cell surface ΔF508-CFTR abundance partially restores the CFTR Cl^−^ channel function (16). Several small molecules called CFTR correctors have been shown to rescue the cell surface expression of ΔF508-CFTR by improving its biosynthetic processing defect in cultured cells (16–19). Correction of the processing defect revealed that rescued (r)ΔF508-CFTR is only partially functional as a Cl^−^ channel, in part because the mutation also alters the post-maturational trafficking and decreases the plasma membrane stability of rΔF508-CFTR (15, 20). The present generation of CFTR correctors does not improve the reduced plasma membrane stability of rΔF508-CFTR, and their clinical efficacy has been limited in patients homozygous for the ΔF508 mutation (16, 21). Hence, understanding regulation of the post-maturational trafficking of CFTR is critical to design effective treatments for CF. Although numerous adaptors have been shown to mediate the post-maturational trafficking of CFTR little is known how this process is regulated (7, 9, 10, 22–30).

The activity of CFTR as a Cl^−^ channel is regulated by protein kinases including protein kinase PKA, PKC, and adenosine monophosphate-stimulated kinase (AMPK) (reviewed in Ref. 31). These kinases target sites in the regulatory domain of CFTR (32, 33). Two of the sites, Ser^737^ and Ser^768^ have a phospho-dependent inhibitory effect on the CFTR channel function (32). Phosphorylation of these inhibitory sites by AMPK inhibits CFTR-mediated Cl^−^ secretion by maintaining the CFTR channels in a closed state (34, 35). Previous work demonstrated that the Ser^737^ site in the recombinant CFTR regulatory domain can be phosphorylated by purified lemur tyrosine kinase 2 (LMTK2) *in vitro* (36). Still, it is unknown whether CFTR is an LMTK2 substrate in airway epithelial cells.

**FIGURE 1.**
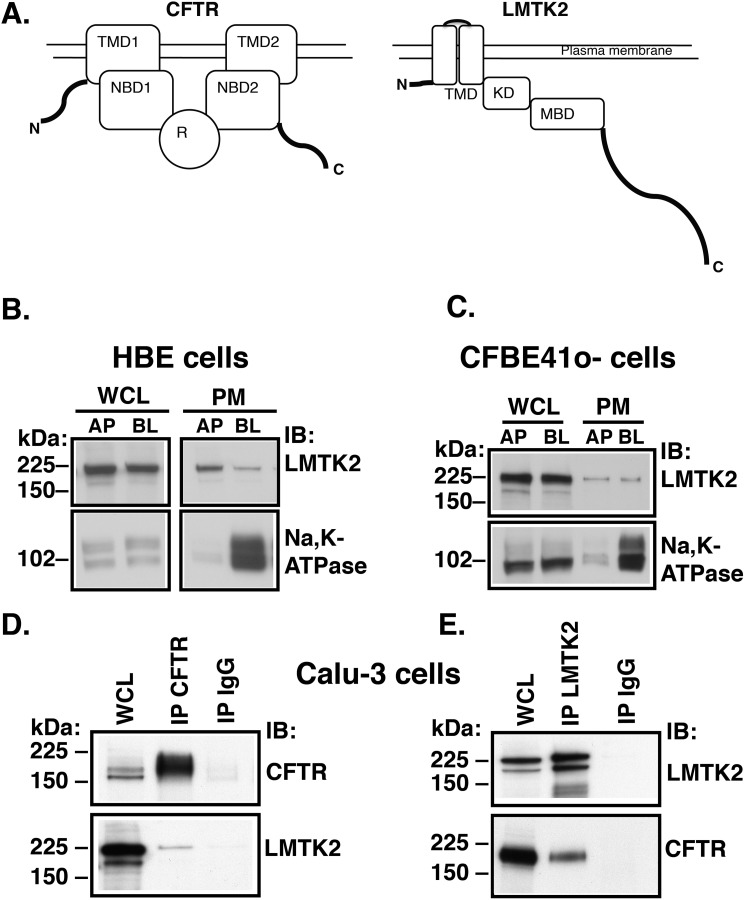
**Experiments demonstrating that LMTK2 localizes at the plasma membrane and co-immunoprecipitates with CFTR in human airway epithelial cells.** *A*, schematic illustration of domain organization in CFTR and LMTK2. CFTR: *TMD*, transmembrane domain; *NBD*, nucleotide binding domain; *R*, regulatory domain. The R contains the phosphor-specific inhibitory Ser^737^ site and other PKA consensus sites. LMTK2: *TMD*, transmembrane domain; *KD*, kinase domain. The residue Lys^168^ located upstream of the Walker A motif is critical for kinase activity (49, 50). *MBD*, myosin binding domain. Amino acid residues 567–773 mediate direct binding to myosin VI (43). *TD*, tail domain. *B* and *C*, immunoblots demonstrating similar distribution of LMTK2 at the plasma membrane of primary differentiated HBE cells and polarized human bronchial epithelial cell model CFBE41o- cells. The apical (*AP*) or basolateral (*BL*) plasma membrane (*PM*) proteins were isolated by domain-selective cell surface biotinylation in monolayers cultured on separate Transwell permeable growth supports. The whole cell lysate (*WCL*) and PM fraction of apically or basolaterally biotinylated monolayer were labeled *AP* and *BL*, respectively. Epithelial cell polarization is demonstrated by the basolateral localization of Na,K-ATPase. LMTK2 was detected at both membrane domains with AP:BL ratio of ∼1:1 when normalized for the corresponding LMTK2 abundance in WCL. WCL represents 5% of BT sample. *D* and *E*, immunoprecipitation experiments demonstrating that LMTK2 and CFTR co-immunoprecipitate in Calu-3 cells. CFTR was immunoprecipitated with the mouse monoclonal antibody M3A7 (IP CFTR, *D*), and LMTK2 was immunoprecipitated with the rabbit polyclonal anti-LMTK2 antibody (IP LMTK2, *E*). Mouse or rabbit non-immune IgGs were used as controls (IP IgG). WCL represents 2% of IP sample. Proteins were separated by SDS-PAGE using 7.5% gels and analyzed by immunobloting (*IB*) as indicated. All experiments were repeated three times from separate cultures with similar results.

LMTK2 also known as kinase/phosphatase/inhibitor-2 (KPI2), brain-enriched kinase (BREK), apoptosis-associated tyrosine kinase (AATYK2), and cyclin-dependent kinase-5 (cdk5/p35) regulated kinase, is a member of the lemur family of membrane-anchored kinases (37–41). Despite the original prediction to be a dual-specificity serine-threonine/tyrosine kinase, studies have shown that purified LMTK2 kinase domain phosphorylates only serine and threonine residues (36, 37, 39). The biological actions of LMTK2 are best described in neuronal and muscle tissues where it plays a role in intracellular trafficking (42–47). LMTK2 forms a regulatory complex with several cytosolic proteins (reviewed in Ref. 48). As shown schematically in Fig. 1*A*, the N-terminal transmembrane domain anchors LMTK2 at the plasma membrane and is followed by the kinase domain (37, 41). The residue Lys^168^ located upstream of the Walker A motif in the catalytic domain is critical for kinase activity (49, 50). The amino acid residues 567–773 mediate direct interaction with myosin VI (43), an actin-based minus-end directed non-conventional motor known to facilitate CFTR endocytosis (26). At the C terminus LMTK2 has a long tail domain that prompted naming the protein after the lemur, a long-tailed Madagascar primate (37). Some of the recognized LMTK2 effects may be cell-type and tissue specific. Thus far nothing is known about the role of LMTK2 in airway epithelial cells. The goals of this study were to determine the localization of LMTK2, whether it associates with and phosphorylates CFTR at residue Ser^737^ (CFTR-Ser^737^) and whether the LMTK2-mediated phosphorylation regulates CFTR endocytic trafficking in human airway epithelial cells. We demonstrate that phosphorylation of CFTR-Ser^737^ mediated by LMTK2 facilitates endocytosis and reduces the cell surface density of CFTR Cl^−^ channels in human airway epithelial cells. Moreover, LMTK2 knockdown increases Cl^−^ secretion mediated by the wild-type (WT) and rΔF508-CFTR. Thus, interfering with LMTK2 phosphorylation of ΔF508-CFTR-Ser^737^ may serve as a novel strategy to improve the cell surface stability of pharmacologically rescued ΔF508-CFTR in CF patients.

## MATERIALS AND METHODS

### 

#### 

##### Cell Lines and Cell Culture

Primary differentiated human bronchial epithelial (HBE) cells (WT-CFTR homozygous) were received from Dr. Joseph Pilewski (Cystic Fibrosis Research Center Epithelial Cell Core at the University of Pittsburgh School of Medicine, Pittsburgh, PA) (51–53). HBE cells were studied because they are considered a gold standard for CFTR research (54); however, these cells are not always available and cannot be passaged, so their use is severely limited. Cells were cultured on human placental collagen-coated Costar Transwell permeable supports (1.12 cm^2^ at a density ∼7 × 10^5^/cm^2^ as previously described and used for experimentation following 6–8 weeks of culture at an air-liquid interface (53).

Human airway epithelial Calu-3 cells that express CFTR endogenously were obtained from the American Type Culture Collection (Manassas, VA). Cells were seeded on Transwell permeable supports (4.67 cm^2^ at density ∼1 × 10^6^) coated with plating medium containing Dulbecco's modified Eagle's medium (DMEM; Invitrogen, Carlsbad, CA) and 10% purified collagen (PureColTM; Advanced Biomatrix, San Diego, CA) and used for experimentation following 3–4 weeks of culture at an air-liquid interface as described previously (27). Calu-3 cells were studied because they express high endogenous levels of CFTR and LMTK2; however, Calu-3 cells are very difficult to transfect.

Human bronchial epithelial cells CFBE41o- stably expressing either WT-CFTR or ΔF508-CFTR cells and parental CFBE41o- (no CFTR transduction), were a generous gift from Dr. J. P. Clancy from the Department of Pediatrics, Cincinnati Children's Hospital, Cincinnati, OH (15, 55). CFBE41o- cells were used because they are isogenic except for CFTR, and they are relatively easy to transfect. CFBE41o- cells express WT-CFTR and ΔF508-CFTR transgenes, thus, studies on these cells do not examine endogenous CFTR. However, stable expression of WT-CFTR in CFBE41o- cells is comparable to endogenous CFTR expression in Calu-3 and HBE cells (9, 26, 53), thus making it a good model of human airway epithelial cells to study regulated CFTR trafficking. CFBE41o- cells were seeded on Transwell permeable supports (4.67 cm^2^ at density ∼1 × 10^6^) coated with plating medium and used for experimentation following 7–9 days of culture at an air-liquid interface as described previously (9, 10). CFBE41o- cells transfected with siRNA were seeded on collagen-coated plastic tissue culture plates (Corning Corporation) and cultured for 96 h to form monolayers. CFBE41o- cells transduced with shRNA were seeded on collagen-coated Snapwell or Transwell permeable supports (Corning Corporation) and cultured for 7–9 days to form polarized monolayers. CFBE41o- cells transfected with plasmid DNA were seeded on collagen-coated plastic tissue culture plates and cultured for 48 h to form monolayers. Fetal bovine serum (FBS) and the selection antibiotic were removed from the media 24 h before experiments to augment cell polarization and cell cycle synchronization (56, 57).

HEK293 cells from the American Type Culture Collection were transfected with plasmid DNA, seeded on plastic tissue culture plates, and used 24 h later for kinase assays. HEK293 cells were also used as a well-accepted model to express recombinant proteins and conduct biochemical studies including the kinase assay.

##### Antibodies and Reagents

The following anti-human CFTR antibodies were used: mouse monoclonal, clone 596 (Cystic Fibrosis Foundation Therapeutics, Inc.; Chapel Hill, NC) and clone M3A7 (Millipore; Billerica, MA), and rabbit polyclonal Ab-737 (Assay Biotechnology Inc. San Francisco, CA). Other antibodies used were rabbit anti-LMTK2 (Sigma-Aldrich) and anti-LMTK2 kinase domain (Cocalico Biologicals Inc., Reamstown, PA), mouse anti-Na,K-ATPase (Millipore), anti-FLAG M2 (Sigma-Aldrich), and anti-ezrin (BD Biosciences, San Jose, CA), and horseradish peroxide-conjugated goat anti-mouse, goat anti-rabbit, secondary antibodies (Bio-Rad). All antibodies were used at the concentrations recommended by the manufacturer. The following reagents were used: Complete Protease Inhibitor Mixture and PhosSTOP phosphatase inhibitor mixture tablets (Roche Applied Sciences, Indianapolis, IN), the adenylate cyclase activator forskolin and the cAMP phosphodiesterase inhibitor IBMX (3-isobutyl-1-methylxanthine) (Sigma-Aldrich), and the inhibitor of protein serine/threonine phosphatases calyculin A (Cell Signaling Technology, Inc.; Danvers, MA).

##### Immunoprecipitation and Immunoblotting

Endogenous CFTR and LMTK2 were immunoprecipitated from Calu-3 or HBE cell lysates by methods described previously (10, 15, 27). Briefly, cultured cells were lysed in an immunoprecipitation (IP) buffer containing 150 mm NaCl, 50 mm Tris, pH 7.4, 1% IGEPAL (Sigma-Aldrich), 5 mm MgCl_2_, 5 mm EDTA, 1 mm EGTA, 30 mm NaF, 1 mm Na_3_VO_4_, and Complete Protease Inhibitor mixture (Roche Applied Sciences, Basel, Switzerland). After centrifugation at 14,000 × *g* for 15 min to pellet insoluble material, the soluble lysates were pre-cleared by incubation with protein G or protein A, as appropriate, conjugated to Sepharose beads (Pierce Chemical Co.) at 4 °C. The pre-cleared lysates were added to the protein G- or protein A-Sepharose beads antibody complexes. CFTR was immunoprecipitated by incubation with the mouse M3A7 antibody and LMTK2 was immunoprecipitated by incubation with the rabbit anti-LMTK2 kinase domain antibody. Non-immune mouse or rabbit IgGs (DAKO North America, Inc., Carpinteria, CA) were used as controls. After washing the protein G- or protein A-Sepharose beads antibody complexes with the IP buffer, immunoprecipitated proteins were eluted by incubation at 85 °C for 5 min in sample buffer (Bio-Rad) containing 100 mm DTT. Immunoprecipitated proteins were separated by SDS-PAGE using 7.5% gels (Bio-Rad) and analyzed by Western blotting. The immunoreactive bands were visualized with Western Lightning Chemiluminescence Reagent Plus (PerkinElmer LAS, Inc., Boston, MA).

##### RNA-mediated Interference

Transfection of CFBE41o- cells with siRNA targeting human LMTK2 gene (siLMTK2; Hs_LMTK2_6 siRNA; Qiagen, Valencia, CA) or the siRNA negative control (siCTRL; AllStars, Qiagen) was conducted using HiPerFect Transfection Reagent (Qiagen) according to the manufacturer's instructions as we previously described (9, 10). For determination of the steady-state plasma membrane abundance of CFTR or CFTR endocytosis, CFBE41o- cells (1.0 × 10^6^) were plated on collagen-coated tissue culture plates and incubated with the optimized transfection mixture containing 10 nm siRNA at 37 °C. The transfection medium was removed after 24 h and cells were cultured on the tissue culture plates until confluent. Under these conditions cells reached confluence at 96 h, and experiments were conducted at 96 h. Silencing the target genes resulted in the corresponding protein depletion by ∼70%. We aimed at such level of silencing to avoid off-target effects that may occur with more dramatic gene silencing.

For short-circuit recordings in Ussing-type chambers CFBE41o- cells (1.0 × 10^6^) were plated on tissue culture plates and incubated with the optimized transfection mixture containing 50 nm siRNA at 37 °C. After 24 h, cells were trypsinized and plated on collagen-coated Snapwell permeable supports and cultured for an additional 6 days to establish polarized monolayers (total 7 days in culture). All experiments were done under the same cell culture conditions to assure similar cellular polarization as well as protein expression and trafficking (10). LMTK2 knockdown under these conditions resulted in the corresponding protein depletion by ∼70%.

Transduction of CFBE41o- cells with shRNAmir targeting the human LMTK2 gene (shLMTK2; V3LHS_345908 or V3LHS_638705) or shRNAmir negative control (RHS4348) in the lentiviral vector pGIPZ with TURBO-GFP reporter (Open Biosystems, Hunstville, AL) was carried at MOI 0.25 according to manufacturer's instructions. Cells transduced with shRNA were selected with puromycin for 5 days, subcultured to collagen-coated Snapwell filters at 1.0 × 10^6^ and cultured in air-liquid interface for 7–9 days to form polarized monolayers.

##### Plasmids and Transient Transfection

The WT-LMTK2Δ-FLAG plasmid was constructed by inserting part of the human LMTK2 sequence coding for the first 600 amino acid residues corresponding to the transmembrane and kinase domain with an engineered C-terminal FLAG into pcDNA3.1 vector (Invitrogen) as previously described (37). The human WT-CFTR was subcloned into pcDNA3.1 vector without a tag (WT-CFTR) (34). To construct the kinase-deficient KM-LMTK2Δ-FLAG fragment the WT-LMTK2Δ-FLAG cDNA was mutated to introduce the K168M substitution and to construct the phosphorylation-deficient CFTR-S737A mutant the WT-CFTR cDNA was mutated using the QuikChange™ II XL site-directed mutagenesis kit (Stratagene) and the KOD Hot Start Kit (Novagene, Darmstadt, Germany). Constructs were sequence verified by ABI PRISM dye terminator cycle sequencing (Applied Biosystems, Foster City, CA). Transfection of cells with plasmids was performed using FuGENE6 (Roche Diagnostics), according to the manufacturer's instructions. CFBE41o- cells transfected with plasmid DNA were seeded on collagen-coated tissue culture plates until confluent. Under these conditions cells reached confluence at 48 h, and experiments were conducted at 48 h.

##### LMTK2 Kinase Assays

Kinase assays were performed as described previously (36). Briefly, HEK293 cells were transfected with WT-LMTK2Δ-FLAG, KM-LMTK2Δ-FLAG or empty pcDNA3.1 vector, and 24 h later, cells were lysed in RIPA buffer without sodium deoxycholate (1% (v/v) Triton X-100, 0.1% (w/v) SDS, 50 mm Tris, pH 7.4, and 150 mm NaCl. LMTK2 was immunoprecipitated with the anti-FLAG M2 antibody (Sigma) and protein G-agarose beads. The beads-antibody-protein complexes were washed three times with RIPA buffer and once with 2× kinase buffer (100 mm Hepes, pH 7.4, 40 mm MgCl_2_, 20% glycerol, 0.02 mg/ml BSA, and 0.02% Brij 35). The kinase assay was performed by incubating the beads for 2 h at 30 °C in a total reaction volume of 50 μl containing 1× kinase buffer, 10 μm ATP, and 20 μm of a peptide corresponding to CFTR residues 733–741 (ERRLSLVPD). A control reaction was performed as above with the exclusion of cell lysates. Extent of phosphorylation was determined using the Kinase-Glo Luminescent Assay (Promega) that assesses the amount of ATP remaining after the reaction and normalized for the amount of ATP in the control reaction.

##### Biochemical Determination of the Plasma Membrane CFTR

The determination of plasma membrane CFTR was performed by domain selective plasma membrane biotinylation in epithelial cell monolayers cultured on permeable growth supports or tissue culture plates using cell membrane impermeable EZ-Link™ Sulfo-NHS-LC-Biotin (Pierce Chemical Co.), followed by cell lysis in buffer containing 25 mm HEPES, pH 8.0, 1% Triton, 10% glycerol, and Complete Protease Inhibitor Mixture (Roche Applied Science), as described previously in detail (58, 59). Biotinylated CFTR was visualized by Western blotting using mouse monoclonal antibody clone 596 and an anti-mouse horseradish peroxidase antibody using the Western Lightning™ Plus-ECL detection system (Perkin Elmer Inc.; Waltham, MA) followed by chemiluminesence. Biotinylated FLAG-tagged LMTK2 fragments were visualized by Western blotting using mouse monoclonal anti-FLAG M2 antibody. Quantification of biotinylated CFTR or the FLAG-tagged LMTK2 was performed by densitometry using exposures within the linear dynamic range of the film. As previously demonstrated with appropriate intracellular controls, under cell culture conditions used in our study only the cell surface proteins are accessible to biotin at 4 °C (9). Western blotting for an intracellular protein, such as ezrin was used as a quality control during each experiment to confirm absence of intracellular proteins in the biotinylated protein samples. Only experiments in which ezrin was not detected in the biotinylated protein samples were included for analysis.

##### Endocytic Assays

Endocytic assays were performed in CFBE41o- cells, as described previously (10, 60). Briefly, the plasma membrane proteins were first biotinylated at 4 °C using cell membrane impermeable and cleavable EZ-Link™ Sulfo-NHS-SS-Biotin (Pierce Chemical Co.). Cells were rapidly warmed to 37 °C for different periods of time after biotinylation and, subsequently, the disulfide bonds on Sulfo-NHS-SS-biotinylated proteins remaining at the plasma membrane were reduced by l-glutathione (GSH; Sigma-Aldrich) at 4 °C. At this point in the protocol, biotinylated proteins reside within the endosomal compartment. Subsequently, cells were lysed, and biotinylated proteins were isolated by streptavidin-agarose beads, eluted into SDS-sample buffer, and separated by 7.5% SDS-PAGE. The amount of biotinylated CFTR at 4 °C and without the 37 °C warming was considered 100%. The amount of biotinylated CFTR remaining at the plasma membrane after GSH treatment at 4 °C and without the 37 °C warming was considered background (<7% compared with the amount of biotinylated CFTR at 4 °C without GSH treatment) and was subtracted from the CFTR biotinylated after warming to 37 °C at each time point. CFTR endocytosis was calculated after subtraction of the background and was expressed as the percent of biotinylated CFTR at each time point after warming to 37 °C compared with the amount of biotinylated CFTR present before warming to 37 °C. We have previously shown that the culture conditions and state of epithelial cell polarization affect CFTR endocytosis in human airway epithelial cells (9, 10, 26, 27). Because endocytic assays were performed in cells cultured on permeable supports or on plastic and in cells transfected with siRNA or plasmid DNA, we determined the time course of CFTR endocytosis for each cell culture and experimental condition and compared with appropriate controls.

##### Short-circuit Recordings

The short circuit currents (*I_SC_*) were measured in Ussing-type chambers (Physiological Instruments; San Diego, CA) as previously described (10, 61). In brief, monolayers of CFBE41o- cells grown on Snapwell permeable supports were mounted in an Ussing-type chamber (Physiologic Instruments) and bathed in solutions (see below) maintained at 37 °C and stirred by bubbling with 5% CO_2_/95% air. Short circuit current (*Isc*) was measured by voltage-clamping the transepithelial voltage across the monolayers to 0 mV with a voltage/current clamp (model VCC MC8, Physiologic Instruments). Transepithelial resistance was measured by periodically applying a 2.5-mV bipolar voltage pulse and was calculated using Ohm's law. The apical bath solution contained (in mm): 115 Na-gluconate, 5 NaCl, 25 NaHCO_3_, 3.3 KH_2_PO_4_, 0.8 K_2_HPO_4_, 1.2 MgCl_2_, 1.2 CaCl_2_, 10 mannitol (pH 7.4). The basolateral bath solution contained (in mm): 120 NaCl, 25 NaHCO_3_, 3.3 KH_2_PO_4_, 0.8 K_2_HPO_4_, 1.2 MgCl_2_, 1.2 CaCl_2_, 10.0 glucose (pH 7.4). A low Cl^−^, high-Na^+^, high-gluconate, apical bath solution was used to prevent cell swelling due to the increased apical Cl^−^ permeability under these conditions, as previously described (10, 61). Following an equilibration period, the baseline *I_SC_* was recorded. Amiloride (10 μm) was added to the apical bath solution to inhibit Na^+^ absorption through ENaC. Subsequently, *Isc* was stimulated with forskolin (10 μm) and IBMX (50 μm) added to the apical and basolateral bath solutions followed by thiazolidonone CFTR inhibitor CFTR_inh_-172 (5 μm) added to the apical bath solution to inhibit CFTR-mediated *Isc.* Data are expressed as forskolin/IBMX stimulated *I*sc, calculated by subtracting the baseline *Isc* from the peak stimulated *Isc*.

##### Data Analysis and Statistics

Statistical analysis of the data were performed using GraphPad Prism version 4.0 for Macintosh (GraphPad Software Inc., San Diego, CA). The means were compared by a two-tailed *t* test. A *p* value <0.05 was considered significant. Data are expressed as mean ± S.E.

## RESULTS

### 

#### 

##### LMTK2 Localizes at the Plasma Membrane in Primary Differentiated Human Bronchial Epithelial Cells

Little is known about the distribution of LMTK2 in human airway epithelial cells. If LMTK2 affects CFTR, it would be expected that it localized, like CFTR, at the apical plasma membrane. Thus, we first examined localization of LMTK2 in primary differentiated HBE cells by domain-selective cell surface biotinylation and Western blotting (53). HBE cells were cultured for 6–8 weeks in air-liquid interface as polarized monolayers. LMTK2 was detected at both the apical and basolateral membrane domain (Fig. 1*B*). Next, we examined localization of LMTK2 in CFBE41o- cells, an immortalized human airway epithelial cell model because unlike HBE these cells are constantly available. Cells were cultured for 7–9 days in air-liquid interface as polarized monolayers. LMTK2 was detected at both the apical and basolateral membrane domain of similar to HBE cells (Fig. 1*C*).

##### Endogenous CFTR and LMTK2 Co-immunoprecipitate in Polarized Human Airway Epithelial Cells

We predicted that LMTK2 could interact with CFTR in polarized human airway epithelial cells. We used Calu-3 cells to study immunoprecipitation because these cells express both CFTR and LMTK2 endogenously, form polarized monolayers, and are constantly available as immortalized cells. Calu-3 cells were cultured for 3–4 weeks on semi-permeable growth supports as polarized monolayers. CFTR was immunoprecipitated with a monoclonal anti-CFTR antibody (M3A7), and LMTK2 was immunoprecipitated with a polyclonal anti-LMTK2 antibody. Western blot analysis demonstrated that endogenous CFTR and LMTK2 co-immunoprecipitate in a reciprocal fashion (Fig. 1, *D* and *E*). Taken together, the co-immunoprecipitation between endogenous CFTR and LMTK2 and their plasma membrane distribution in primary human airway epithelial cells suggest a physical association between CFTR and LMTK2 within the apical plasma membrane.

##### LMTK2 Knockdown Increases Plasma Membrane Abundance of CFTR in Human Airway Epithelial Cells

**FIGURE 2.**
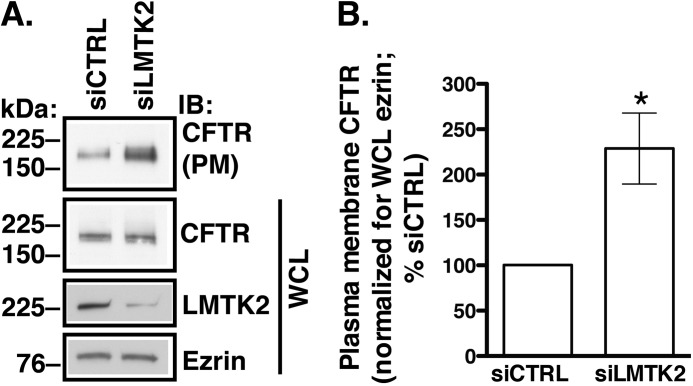
**Biotinylation experiments demonstrating that LMTK2 knockdown increases CFTR abundance at the plasma membrane in CFBE41o- cells.** Immunoblots (*A*) and summary of experiments (*B*). CFBE41o- cells stably expressing WT-CFTR were transfected with 10 nm siRNA specific for LMTK2 (siLMTK2) or the siRNA-negative control (siCTRL) and cultured on collagen-coated tissue culture plates for 96 h to form monolayers. LMTK2 abundance in whole cell lysates (*WCL*) was reduced by siLMTK2 to 30.0 ± 6.7% (*p* < 0.05 *versus* siCTRL, *n* = 5, mean ± S.E.). By contrast siLMTK2 did not change the abundance of ezrin. siLMTK2 increased the steady-state plasma membrane (*PM*) abundance of CFTR without affecting WCL CFTR indicating that LMTK2 affects the post-maturational trafficking of CFTR at the plasma membrane without affecting its biosynthetic processing. Plasma membrane proteins were isolated by cell surface biotinylation, and the plasma membrane CFTR was normalized for WCL ezrin. Ezrin expression was used as a loading control. WCL represents 5% of BT sample. *, *p* < 0.05 *versus* siCTRL. Five experiments/group. Error bars, S.E.

We hypothesized that LMTK2 controls CFTR trafficking at the plasma membrane in human airway epithelial cells. To test this hypothesis, LMTK2 expression was knocked down in CFBE41o- cells stably expressing WT-CFTR by RNA interference. We used CFBE41o- cells because these immortalized cell lines localize LMTK2 at the plasma membrane similar to the primary HBE cells as shown in Fig. 1. Moreover CFBE41o- cells serve as good models for CFTR trafficking (9, 10, 15, 61, 62). Cells were transfected with siRNA specific for the human LMTK2 sequence (siLMTK2) or with a siRNA control (siCTRL) and cultured on collagen-coated tissue culture plates for 96 h as monolayers (Fig. 2). The siLMTK2 reduced LMTK2 protein abundance in whole cell lysates by ∼70% without decreasing the levels of CFTR or ezrin (Fig. 2*A*). As determined by cell surface biotinylation and Western blotting, knockdown of LMTK2 increased the steady-state plasma membrane abundance of CFTR (Fig. 2, *A* and *B*).

##### LMTK2 Knockdown Increases the CFTR-mediated Isc Across Polarized Human Airway Epithelial Cells

**FIGURE 3.**
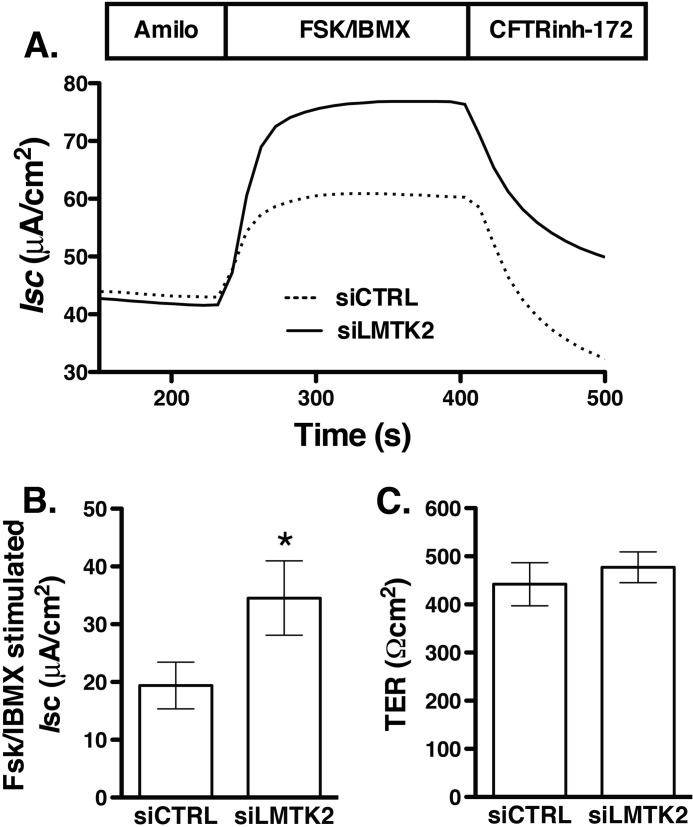
**Ussing chamber experiments demonstrating that LMTK2 knockdown increases CFTR mediated *I*sc across CFBE41o- monolayers.** CFBE41o- cells stably expressing WT-CFTR were plated on tissue culture plates and incubated with the optimized transfection mixture containing 50 nm of siRNA specific for LMTK2 (siLMTK2) or the siRNA negative control (siCTRL). After 24 h, cells were trypsinized and plated on collagen-coated Snapwell permeable supports and cultured for an additional 6 days to establish polarized monolayers (total 7 days in culture). CFBE41o- cells were bathed in solutions with apical-to-basolateral Cl^−^ gradient in the presence of amiloride (Amilo, 50 μm) in the apical bath solution to inhibit Na^+^ absorption through ENaC. *I*sc was stimulated with forskolin (FSK, 20 μm) and IBMX (50 μm) added to the apical and basolateral bath solution. Thiazolidonone CFTR_inh_-172 (5 μm) was added to the apical bath solution to inhibit CFTR-mediated *Isc.* Data are expressed as net stimulated *I*sc, calculated by subtracting the baseline *Isc* from the peak stimulated *Isc*. siCTRL did not affect the forskolin/IBMX-stimulated *I*_sc_ across CFBE41o-cells compared with the non-transfected cells (data not shown). Representative experiment (*A*) and summary of data (*B*) demonstrating that LMTK2 knockdown increased the forskolin/IBMX-stimulated *I*sc across CFBE41o- cells. siLMTK2 did not change the transepithelial resistance across the monolayers (*C*). *, *p* < 0.05 *versus* siCTRL. 9 monolayers/group from 2 different cultures. Error bars, S.E.

CFTR mediated Cl^−^ secretion is determined by the activity and number of CFTR Cl^−^ channels at the plasma membrane. Because LMTK2 knockdown increased the plasma membrane abundance of CFTR, we analyzed the functionality of the rescued fraction, predicting that it would also increase CFTR-mediated Cl^−^ secretion. siLMTK2 increased the forskolin/IBMX-stimulated *I*sc across CFBE41o- monolayers (Fig. 3). These results are consistent with the above data that siLMTK2 increased CFTR abundance at the plasma membrane and suggest that endogenous LMTK2 decreases CFTR-mediated Cl^−^ secretion, at least in part by decreasing density of the CFTR Cl^−^ channels at the plasma membrane in human airway epithelial cells.

##### LMTK2 Knockdown Decreases CFTR Endocytosis in Human Airway Epithelial Cells

**FIGURE 4.**
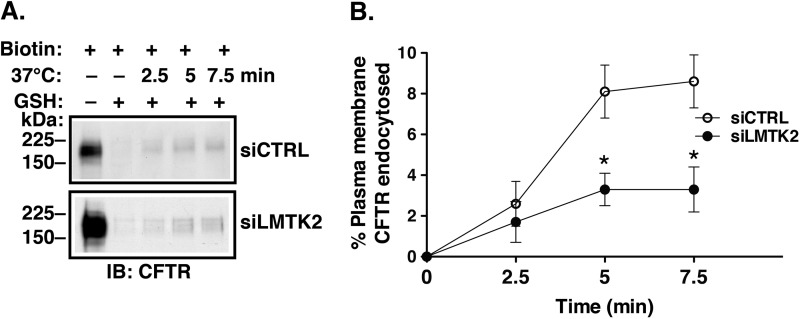
**Endocytic assays demonstrating that LMTK2 knockdown decreased CFTR endocytosis in CFBE41o- cells.** CFBE41o- cells stably expressing WT-CFTR were transfected with 10 nm siLMTK2 or the siRNA control (*siCTRL*) and cultured on collagen-coated tissue culture plates for 96 h to form monolayers. We previously showed that siCTRL does not affect CFTR endocytosis under similar cell culture conditions (10). The plasma membrane proteins were first biotinylated at 4 °C using cell membrane impermeable and cleavable EZ-Link™ Sulfo-NHS-SS-Biotin (Biotin). Cells were rapidly warmed to 37 °C for different periods of time after biotinylation, and, subsequently, the disulfide bonds on Sulfo-NHS-SS-biotinylated proteins remaining at the plasma membrane were reduced by l-glutathione (*GSH*) at 4 °C. At this point in the protocol, biotinylated proteins reside within the endosomal compartment. The amount of biotinylated CFTR at 4 °C and without the 37 °C warming was considered 100%. The amount of biotinylated CFTR remaining after the GSH treatment at 4 °C without warming to 37 °C was considered background and was subtracted from the amount of biotinylated CFTR remaining after warming to 37 °C at each time point. CFTR endocytosis was calculated after subtraction of the background and was expressed as the percent of CFTR remaining biotinylated before and after warming to 37 °C. Immunoblots (*A*) and summary of experiments (*B*) demonstrating that siLMTK2 decreased CFTR endocytosis. Please, note the increased abundance of CFTR before endocytosis (*A*, the first lane) in siLMTK2 cells compared with siCTRL consistent with the increased plasma membrane abundance of CFTR at steady-state demonstrates in Fig. 2. Ezrin expression in WCL was used as a loading control (data not shown). *, *p* < 0.05 *versus* siCTRL. 4–6 experiments/group. Error bars, S.E.

Increased CFTR abundance at the plasma membrane could result from decreased endocytosis or increased recycling of CFTR. Endocytic assays were conducted to determine the mechanism of increased CFTR abundance at the plasma membrane due to LMTK2 knockdown. CFBE41o- cells stably expressing WT-CFTR were transfected with siRNA specific for human LMTK2 sequence (siLMTK2) or with a siRNA control (siCTRL) and cultured on collagen-coated tissue culture plates for 96 h as monolayers. Endocytic assays were performed by the GSH protection assay (10, 60). As illustrated in Fig. 4, siLMTK2 decreased CFTR endocytosis compared with siCTRL. Attenuation of CFTR endocytosis is consistent with increased steady-state plasma membrane abundance of CFTR in LMTK2 knockdown cells. Together, these data demonstrate that LMTK2 facilitates CFTR endocytosis in polarized human airway epithelial cells.

##### LMTK2 Phosphorylates CFTR in Human Airway Epithelial Cells

**FIGURE 5.**
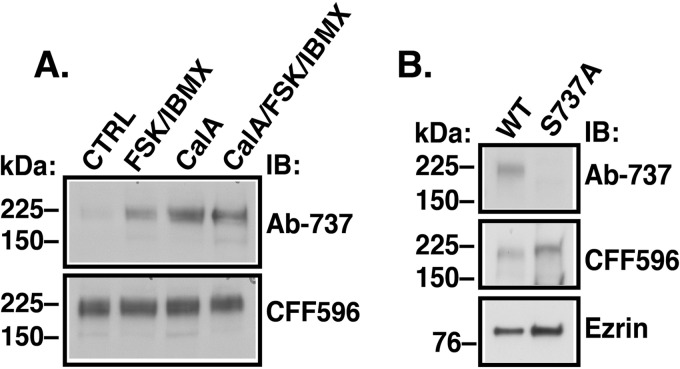
**Immunoblots demonstrating that the anti-CFTR phosphosite antibody Ab-737 recognizes specifically phosphorylation the Ser^737^ site.** *A*, CFBE41o- cells stably expressing WT-CFTR were treated with IBMX (1 mm) and forskolin (FSK, 20 μm) added to the apical and basolateral medium at 37 °C for 10 min before experiments to raise the intracellular cAMP levels and promote PKA-mediated phosphorylation (34). To increase the phosphorylated CFTR fraction we attenuated serine/threonine protein phosphatases by treating cells with calyculin A (CalA, 50 nm) added to the basolateral medium at 37 °C for 15 min before experiments (63, 64). Low level of CFTR was detected by the phosphosite antibody Ab-737 in WCL of the vehicle control (CTRL)-treated cells. By contrast, the phosphosite antibody detected more CFTR after treatment with either forskolin/IBMX or calyculin A or both. *B*, parental CFBE41o- cells were transfected with the WT-CFTR (WT) or the mutant CFTR-S737A (S737A). Unlike the WT-CFTR, the CFTR-S737A was not detected by the antibody Ab-737 in WCL. Antibody CFF596 was used to detect the total CFTR. Ezrin expression was used as a loading control. Experiments were repeated at least three times from different cultures with similar results.

To determine whether CFTR is an LMTK2 substrate in airway epithelial cells, CFBE41o- cells stably expressing WT-CFTR and cultured as polarized monolayers on permeable growth supports for 7–9 days were treated with IBMX (1 mm) and forskolin (20 μm) added to the apical and basolateral medium at 37 °C for 10 min before experiments to raise the intracellular cAMP levels and promote PKA-mediated phosphorylation (34). To further enhance phosphorylation of CFTR we treated cells with the cell-permeable phosphatase inhibitor calyculin A (50 nm), added to the basolateral medium at 37 °C for 15 min before experiments (63, 64). Low levels of CFTR were detected by the phosphosite specific antibody Ab-737 in the vehicle control (CTRL)-treated cells, representing baseline levels of CFTR phosphorylated at Ser^737^ (CFTR-pS^737^; Fig. 5*A*). By contrast, CFTR phosphorylation was greatly enhanced after the forskolin/IBMX and calyculin A treatment (Fig. 5*A*). To demonstrate specificity of the phosphosite antibody, parental CFBE41o- cells were transiently transfected with WT-CFTR or CFTR with the S737A substitution (CFTR-S737A) inactivating the phosphorylation site (31). Even with calyculin A treatment of the cells, the CFTR-S737A mutant was not detected by antibody Ab-737, unlike the WT-CFTR (Fig. 5*B*). Together, these results demonstrate that the phosphosite antibody Ab-737 recognizes specifically the CFTR-pS^737^.

LMTK2 was shown to phosphorylate CFTR Ser^737^ *in vitro* (36). We assessed the effects of altering the LMTK2 kinase on CFTR phosphorylation in CFBE41o-. The residue Lys^168^ located upstream of the Walker A motif in the catalytic domain of LMTK2 is predicted to be critical for catalysis (49, 50). Site-directed mutagenesis was used to produce the K168M substitution in LMTK2 kinase domain. We expressed an LMTK2 fragment containing the first 600 amino acids including the transmembrane and kinase domain with an engineered C-terminal FLAG epitope tag (WT-LMTK2Δ-FLAG) and confirmed it was expressed at the plasma membrane, similar to endogenous LMTK2 (data not shown). Next, we expressed the K168M version (KM-LMTK2Δ-FLAG). Expressing the truncated LMTK2 fragments WT-LMTK2Δ-FLAG and KM-LMTK2Δ-FLAG in human airway epithelial cells eliminated the interactions mediated by the myosin VI binding domain and tail domain in the full-length LMTK2 and thus, allowed us to specifically examine effects mediated by the LMTK2 transmembrane and kinase domain.

**FIGURE 6.**
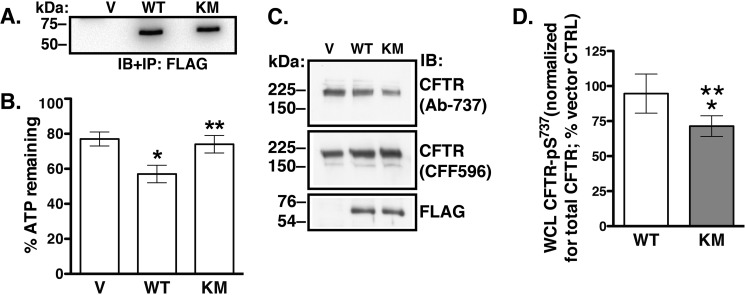
**Experiments demonstrating that the LMTK2-K168M mutation reduces CFTR phosphorylation *in vitro* and in CFBE41o- cells.** Immunoblot (*A*) and summary (*B*) of *in vitro* phosphorylation assays. The vector control (*V*), WT-LMTK2Δ-FLAG (*WT*), and KM-LMTK2Δ-FLAG (*KM*) were immunoprecipitated (*IP*) from the HEK293 cell lysates and immunoblotted (*IB*) using the anti-FLAG M2 antibody. The immunoprecipitated protein complexes were used to *in vitro* phosphorylate a peptide encompassing CFTR amino acids 733–741. The extent of phosphorylation was determined using the Kinase-Glo Luminescent Assay that assesses the amount of ATP remaining after the reaction and normalized for the amount of ATP in the control reaction. The ATP level decreased in the reaction containing the WT-LMTK2Δ-FLAG indicating that LMTK2 phosphorylated the CFTR-derived peptide. By contrast, ATP levels were similar in the reactions containing either the KM-LMTK2Δ-FLAG or vector control indicating that the K168M mutation decreased phosphorylation of the CFTR derived peptide. Immunoblots (*C*) and summary of data (*D*) demonstrating that the kinase deficient LMTK2 fragment KM-LMTK2Δ-FLAG (KM) decreases CFTR-pS^737^ abundance in whole cell lysate (WCL) of CFBE41o- cells stably expressing WT-CFTR. Forty-eight hours after transfecting the WT-LMTK2Δ-FLAG (WT), KM-LMTK2Δ-FLAG (KM), or vector control (V) cells were treated with calyculin A and the WCL abundance of CFTR-pS^737^ detected with phosphosite antibody Ab-737 was normalized for the total CFTR detected with antibody CFF596. The WT-LMTK2Δ-FLAG fragment did not increase the WCL CFTR-pS^737^ compared with vector control. By contrast, the KM-LMTK2Δ-FLAG mutant decreased CFTR-pS^737^ in WCL compared with vector control or the WT-LMTK2Δ-FLAG. Immunoblotting with anti-FLAG antibody demonstrates similar expression of the LMTK2 fragments. *, *p* < 0.05 *versus* vector control. **, *p* < 0.05 *versus* WT-LMTK2Δ-FLAG. Six experiments/group (*A* and *B*) or four experiments/group (*C* and *D*). Error bars, S.E.

To ascertain that the K168M mutation impairs LMTK2 kinase activity, we transfected HEK293 cells with the WT-LMTK2Δ-FLAG and KM-LMTK2Δ-FLAG or empty pcDNA3.1 vector and the resulting protein fragments were immunoprecipitated with anti-FLAG M2 antibody. The immunoprecipitated protein complexes were used to *in vitro* phosphorylate a peptide encompassing CFTR amino acids 733–741. Extent of phosphorylation was determined using the Kinase-Glo Luminescent Assay that assesses the amount of ATP remaining after the reaction normalized for the amount of ATP in the control reaction as described in “Materials and Methods.” ATP levels decreased in the reaction containing the WT-LMTK2Δ-FLAG indicating that LMTK2 phosphorylated the CFTR-derived peptide (Fig. 6, *A* and *B*). By contrast, ATP levels were similar in the reaction containing either the KM-LMTK2Δ-FLAG or vector control indicating that the K168M mutation impaired phosphorylation of the CFTR-derived peptide (Fig. 6, *A* and *B*). These results confirm the prediction that the residue Lys^168^ located in the catalytic domain of LMTK2 is critical for catalysis.

Next, we assessed phosphorylation of CFTR-Ser^737^ using the phosphosite antibody Ab-737. CFBE41o- cells stably expressing WT-CFTR were transfected with WT-LMTK2Δ-FLAG, KM-LMTK2Δ-FLAG, or vector control (V). Forty-eight hours after transfection cells were treated with calyculin A (50 nm) added to the basolateral medium at 37 °C for 15 min before experiments. CFTR-pS^737^ detected by immunoblotting with antibody Ab-737 was normalized for total CFTR detected with antibody CFF596. CFTR-pS^737^ abundance was decreased in cells expressing KM-LMTK2Δ-FLAG compared with WT-LMTK2Δ-FLAG or vector control (Fig. 6, *C* and *D*). Our conclusion was that the LMTK2 fragment with inactive kinase KM-LMTK2Δ-FLAG attenuated phosphorylation of CFTR-Ser^737^ because it competed with endogenous LMTK2 for phospohorylation of the CFTR-Ser^737^ site in CFBE41o- cells.

##### LMTK2-mediated Phosphorylation of CFTR Facilitates Endocytosis and Attenuates CFTR Abundance at Plasma Membrane

**FIGURE 7.**
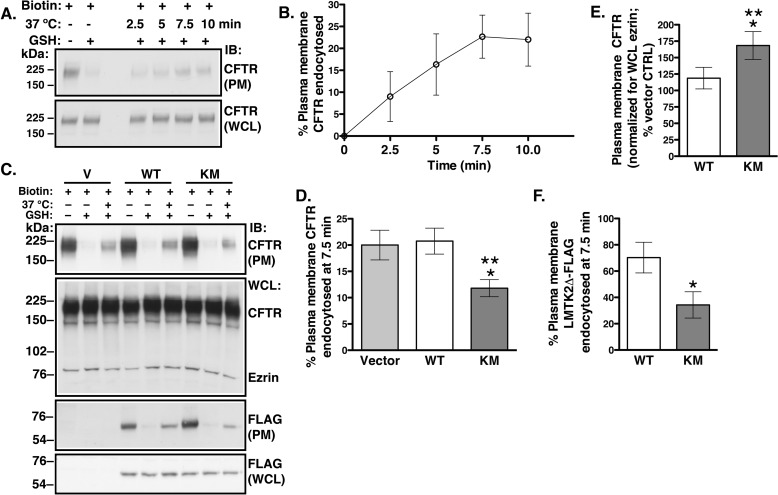
**Experiments demonstrating effects of the LMTK2 kinase on endocytosis and the steady-state plasma membrane abundance of CFTR in CFBE41o- cells.** CFBE41o- cells stably expressing WT-CFTR were transfected with the WT-LMTK2Δ-FLAG (*WT*), KM-LMTK2Δ-FLAG (*KM*), or vector control (*V*) and cultured for 48 h on collagen-coated tissue culture plates to form monolayers. Immunoblots (*A*) and summary of data (*B*) demonstrating that CFTR endocytosis was linear up to 7.5 min under the cell culture conditions. The plasma membrane (*PM*) and whole cell lysate (*WCL*) CFTR was detected with antibody CFF596. CFTR endocytosis was calculated as described in “Materials and Methods” and the Fig. 4 legend. Based on the above results, we examined the effects of the WT-LMTK2Δ-FLAG and KM-LMTK2Δ-FLAG at the 7.5-min time representing the linear portion of CFTR endocytosis. Immunoblots (*C*, *IB*: *CFTR*) and summary of results (*D*) demonstrating that the KM-LMTK2Δ-FLAG decreased CFTR endocytosis when compared with the WT-LMTK2Δ-FLAG or vector control. *E*, summary of data demonstrating that the KM-LMTK2Δ-FLAG increased CFTR abundance at the plasma membrane at steady-state when compared with the WT-LMTK2Δ-FLAG or vector control (*i.e.* endogenous LMTK2). The steady-state plasma membrane CFTR was calculated from the amount of biotinylated CFTR at time zero during the endocytic assays (before warming to 37 °C and without GSH treatment). Immunoblots (*C*, *IB*: *FLAG*) and summary of data (*F*) examining the plasma membrane abundance and endocytosis of the WT-LMTK2Δ-FLAG and KM-LMTK2Δ-FLAG fragments. Compared with the WT-LMTK2Δ-FLAG, the KM-LMTK2Δ-FLAG fragment was more abundant at the plasma membrane due to decreased endocytosis. Endocytosis of the FLAG-tagged fragments was calculated as described for CFTR. The WCL expression of WT-LMTK2Δ-FLAG or KM-LMTK2Δ-FLAG was used, when appropriate as a loading control to account for small differences in the expression levels of the transiently transfected protein fragments. *, *p* < 0.05 *versus* vector control (*V*). **, *p* < 0.05 *versus* WT-LMTK2Δ-FLAG. 3–4 experiments/group. Error bars, S.E.

The above data have shown that LMTK2 facilitates CFTR endocytosis and that LMTK2 phosphorylates CFTR-Ser^737^ in human airway epithelial cells. Studies were conducted to determine whether CFTR endocytosis is regulated by the LMTK2 phosphorylation of CFTR-Ser^737^. Endocytic assays were performed to examine the effects of WT-LMTK2Δ-FLAG and KM-LMTK2Δ-FLAG on CFTR endocytosis. We determined the time course of CFTR endocytosis in CFBE41o- cells stably expressing WT-CFTR cultured for 48 h as monolayers on collagen-coated tissue culture plates. CFTR endocytosis reached maximum at 7.5 min (Fig. 7, *A* and *B*). Thus, in subsequent experiments we examined CFTR endocytosis at the 7.5-min time point. Differences in the magnitude and maximum of CFTR endocytosis in CFBE41o- cells cultured as polarized monolayers on permeable growth supports in Fig. 4 and CFBE41o- cells cultured as monolayers on tissue culture plates in Fig. 7 are consistent with the know effects of epithelial cell culture conditions on endocytic trafficking (65–67). CFBE41o- cells were transfected with the WT-LMTK2Δ-FLAG, KM-LMTK2Δ-FLAG, or vector control (V) and endocytic assays were performed 48 h after transfection when the cells formed monolayers. As expected from the effects on CFTR phosphorylation, the KM-LMTK2Δ-FLAG decreased CFTR endocytosis compared with either the WT-LMTK2Δ-FLAG or vector control (Fig. 7, *C* and *D*). Moreover, the KM-LMTK2Δ-FLAG increased the steady-state plasma membrane CFTR abundance when compared with the WT-LMTK2Δ-FLAG or vector control (Fig. 7, *C* and *E*). Inhibition of CFTR endocytosis is consistent with increased CFTR abundance at plasma membrane in the KM-LMTK2Δ-FLAG-transfected cells. Because expression of the WT-LMTK2Δ-FLAG did not increase phosphorylation of CFTR (Fig. 6, *C* and *D*) these conditions were not expected to alter endocytosis or the plasma membrane abundance of CFTR compared with vector control. Indeed, the WT-LMTK2Δ-FLAG had no effect on endocytosis or the plasma membrane abundance of CFTR compared with vector control (Fig. 7, *C--E*). Together, the above results demonstrate that the LMTK2 phosphorylation of CFTR-Ser^737^ facilitates CFTR endocytosis and reduces CFTR abundance at the plasma membrane.

Nothing is known about membrane trafficking of LMTK2 in human airway epithelial cells. Thus, to gain insight, we examined the plasma membrane abundance and endocytic uptake of the LMTK2 fragments in CFBE41o- cells. Approximately 70% of the WT-LMTK2Δ-FLAG was internalized in 7.5 min (Fig. 7, *C* and *F*). By contrast, internalization of the KM-LMTK2Δ-FLAG was decreased by 50%. Compared with the WT-LMTK2Δ-FLAG, the KM-LMTK2Δ-FLAG fragment was more abundant at the plasma membrane due to decreased endocytosis (Fig. 7, *C* and *F*). These data demonstrate for the first time the dynamic, kinase domain-dependent, spatial, and temporal regulation of LMTK2 distribution between the cell surface and endocytic compartments, consistent with the role of LMTK2 in endocytic trafficking in human airway epithelial cells.

##### The Phosphorylation-deficient CFTR-S737A Mutant is More Abundant at the Plasma Membrane Due to Decreased Endocytosis

**FIGURE 8.**
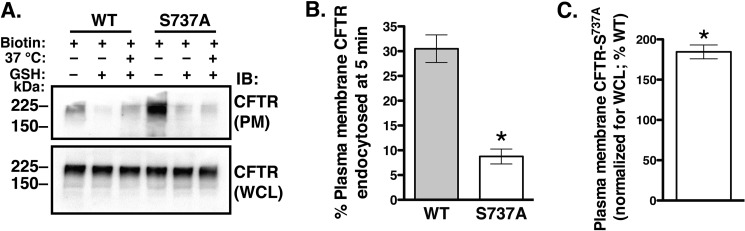
**Experiments demonstrating that compared with WT-CFTR the phosphorylation-deficient mutant CFTR S737A has increased plasma membrane abundance at steady-state and decreased endocytosis in CFBE41o- cells.** Parental CFBE41o- cells were transfected with the WT-CFTR (*WT*) or the CFTR-S737A mutant (S737A), and cells were cultured on collagen-coated tissue culture plates for 48 h to form monolayers. Endocytosis of the transiently transfected WT-CFTR in parental CFBE41o- cells was linear up to 5 min (data not shown), thus data are reported at the 5-min time point. Immunoblots (*A*) and summary of data (*B*) demonstrating that the S737A mutation decreased CFTR endocytosis. The amount of biotinylated WT-CFTR or CFTR-S737A remaining after the GSH treatment at 4 °C without warming to 37 °C was considered background and was subtracted from the amount of biotinylated WT-CFTR or CFTR-S737A remaining after warming to 37 °C. Endocytosis of WT-CFTR or CFTR-S737A was calculated after subtracting the background (see above) and was expressed as the percent of WT-CFTR or CFTR-S737A remaining biotinylated before and after warming to 37 °C. WT-CFTR and CFTR-S737A was detected with antibody CFF596. *C*, summary of data showing plasma membrane (*PM*) CFTR abundance at steady-state. The steady-state plasma membrane CFTR was calculated from the amount of biotinylated CFTR at time 0 during the endocytic assays (before warming to 37 °C and without GSH treatment). The whole cell lysate (*WCL*) expression of WT-CFTR or CFTR-S737A mutant detected with antibody CFF596 was used as a loading control to account for small differences in the expression levels of the transiently transfected proteins. *, *p* < 0.05 *versus* WT-CFTR. Three experiments/group. Error bars, S.E.

If the LMTK2 mediated phosphorylation of CFTR-Ser^737^ induces CFTR endocytosis, eliminating the phosphorylation site by the S737A substitution should reduce CFTR endocytosis. Parental CFBE41o- cells were transfected with WT-CFTR or the CFTR-S737A mutant. We determined the time course of WT-CFTR endocytosis in parental CFBE41o- cells cultured for 48 h as monolayers on collagen-coated tissue culture plates. CFTR endocytosis reached maximum at 5 min (data not shown). Thus, in subsequent experiments we examined CFTR endocytosis at the 5-min time point. The S737A mutation attenuated CFTR endocytosis and increased the plasma membrane abundance of CFTR (Fig. 8). Together, the above results demonstrate that the LMTK2 phosphorylation of CFTR-Ser^737^ facilitates CFTR endocytosis and reduces the plasma membrane abundance of CFTR in human airway epithelial cells.

##### LMTK2 Knockdown Facilitates the Corrector-mediated Functional Rescue of ΔF508-CFTR

**FIGURE 9.**
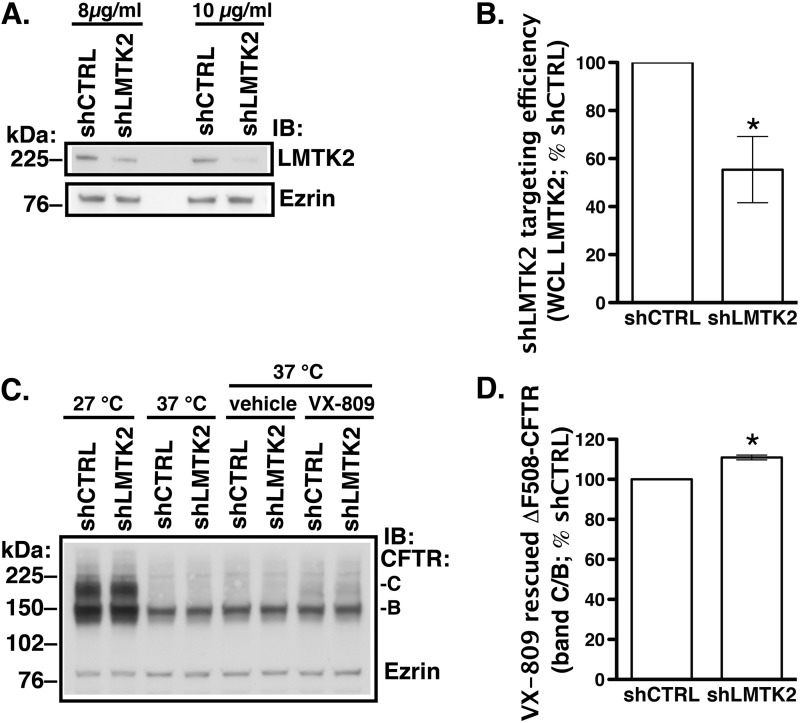
**Experiments demonstrating effects of LMTK2 knockdown on ΔF508-CFTR abundance in whole cell lysates (*WCL*) of polarized CFBE41o**- **cells.** CFBE41o- cells stably expressing ΔF508-CFTR were transduced with shRNA against the human LMTK2 gene (shLMTK2) or the shRNA negative control (shCTRL). Transduced cells were selected with puromycin. The concentration of puromycin was titrated to specifically select cells transduced with a target copy number of viral particles producing LMTK2 knockdown without off-target effects. *A*, immunoblots showing improved LMTK2 knockdown in cells selected with 10 μg/ml of puromycin compared with 8 μg/ml without affecting expression of the cytoskeletal protein ezrin. Cells selected with 10 μg/ml of puromycin for 5 days were subcultured to collagen-coated Transwell filters at 1.0 × 10^6^ and cultured in air-liquid interface for 7–9 days to form polarized monolayers in medium supplemented with the maintenance concentration of puromycin (2 μg/ml). *B*, summary of data showing LMTK2 knockdown in polarized monolayers before experiments. VX-809 (10 μm) or low temperature (27 °C) was used for 48 h before experiments to facilitate the biosynthetic processing and rescue the abundance of the mature, fully glycosylated, plasma membrane associated band C of ΔF508-CFTR. *C*, immunoblot demonstrating that VX-809 increased the ΔF508-CFTR band C abundance compared with vehicle control and the effect was partial compared with low temperature. LMTK2 knockdown increased the steady-state abundance of the VX-809 rescued ΔF508-CFTR band C (*C* and *D*). LMTK2 knockdown did not increase the steady-state abundance of the partially glycosylated ΔF508-CFTR band B (*C*). LMTK2 knockdown did not increase the abundance of the ΔF508-CFTR band C in the absence of VX-809 rescue, indicating that LMTK2 did not facilitate the biosynthetic processing of ΔF508-CFTR (*C*). Ezrin expression was used as a loading control. *, *p* < 0.05 *versus* shCTRL. Four experiments/group. Error bars, S.E.

**FIGURE 10.**
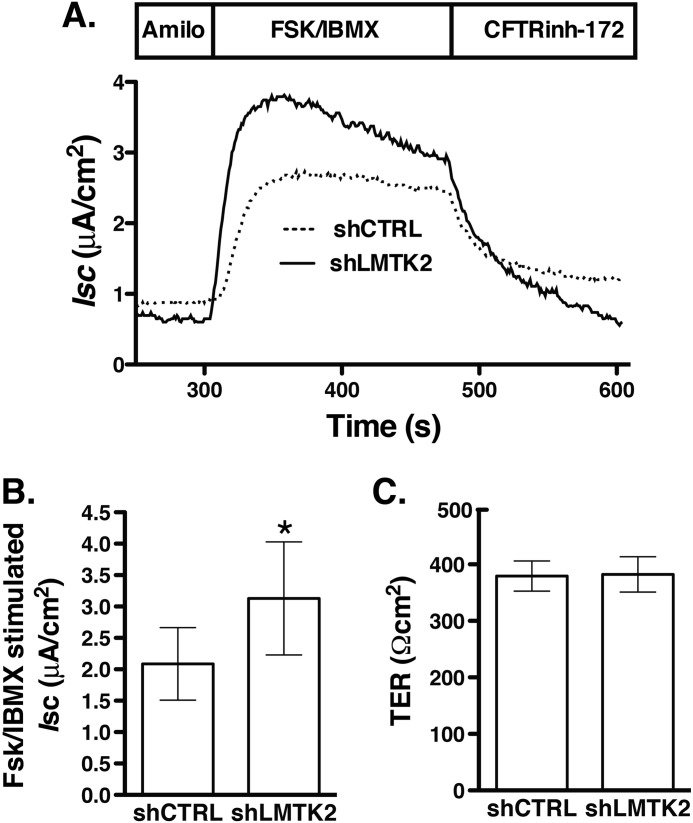
**Ussing chamber experiments demonstrating that LMTK2 knockdown facilitates the CFTR-mediated *I*sc across CFBE41o- monolayers expressing ΔF508-CFTR rescued with VX-809.** CFBE41o- cells stably expressing ΔF508-CFTR and transduced with shRNA against the human LMTK2 gene (shLMTK2) or the shRNA negative control (shCTRL) as described in Fig. 10 were cultured on collagen-coated Snapwell filters at 1.0 × 10^6^ in air-liquid interface for 7–9 days. VX-809 (10 μm) was used for 48 h before experiments to facilitate the biosynthetic processing and rescue the abundance of ΔF508-CFTR band C. CFBE41o- cells were bathed in solutions with apical-to-basolateral Cl^−^ gradient in the presence of amiloride (Amilo, 50 μm) in the apical bath solution to inhibit Na^+^ absorption through ENaC. *I*sc was stimulated with forskolin (FSK, 20 μm) and IBMX (50 μm) added to the apical and basolateral bath solution. Thiazolidonone CFTRinh-172 (5 μm) was added to the apical bath solution to inhibit CFTR-mediated *I*sc. Data are expressed as net stimulated *I*sc, calculated by subtracting the baseline *I*sc from the peak stimulated *I*sc. shCTRL did not affect the forskolin/IBMX-stimulated *I*sc across CFBE41o- cells compared with the non-transfected cells (data not shown). Representative experiment (*A*) and summary of data (*B*) demonstrating that LMTK2 knockdown increased the forskolin/IBMX-stimulated *I*sc across CFBE41o-cells after the VX-809 rescue. shLMTK2 did not change the transepithelial resistance across cell monolayers (*C*). *, *p* < 0.05 *versus* siCTRL. 16–17 monolayers/group from two different cultures. Error bars, S.E.

Although the small molecule VX-809 partially corrected the biosynthetic processing defect of ΔF508-CFTR in cultured cells, it had disappointing effects as a monotherapy in patients homozygous for the ΔF508 mutation (17, 21). Rescued ΔF508-CFTR demonstrates compromised plasma membrane stability due to altered post-maturational trafficking. Hence, correction of the trafficking defect may help to stabilize the mutant CFTR at the cell surface after the biosynthetic rescue (15, 20). Because LMTK2 knockdown increased the cell surface density of CFTR by inhibiting the endocytic uptake of CFTR, we examined whether this manipulation would facilitate the VX-809 mediated rescue of ΔF508-CFTR. CFBE41o- cells expressing ΔF508-CFTR were transduced with shRNA against LMTK2 (shLMTK2) or the shRNA negative control (shCTRL). Transduced cells were selected with puromycin. The concentration of puromycin was titrated to select cells transduced with a target copy number of viral particles producing LMTK2 knockdown without off-target effects (Fig. 9, *A* and *B*). VX-809 partially rescued the biosynthetic processing defect of ΔF508-CFTR demonstrated by appearance of the mature, fully glycosylated CFTR band C (Fig. 9*C*). Compared with control (shCTRL), LMTK2 knockdown increased band C abundance and the forskolin/IBMX-stimulated *I*sc across CFBE41o- monolayers (Figs. 9*D* and 10). These data indicate that LMTK2 knockdown facilitated the VX809-mediated rescue of ΔF508-CFTR.

## DISCUSSION

The present study discovered that in human airway epithelial cells CFTR endocytosis is regulated by the LMTK2-mediated phosphorylation of CFTR-Ser^737^ that decreases the cell surface density of CFTR Cl^−^ channels and inhibits CFTR-mediated Cl^−^ secretion. The clinical implications of our findings are that regulating LMTK2 phosphorylation of CFTR may serve as a novel approach to stabilize CFTR at the cell surface. This approach may be particularly useful to stabilize pharmacologically rescued ΔF508-CFTR in CF patients.

Several lines of evidence support these conclusions. First, endogenous LMTK2 accumulated at the apical membrane domain of polarized human airway epithelial cells, including primary differentiated HBE cells and co-immunoprecipitated with CFTR, indicating that LMTK2 and CFTR physically associate within this membrane domain (Fig. 1). Second, inactivation by mutagenesis of the kinase activity in the truncated LMTK2 fragment KM-LMTK2Δ-FLAG inhibited phosphorylation of CFTR-Ser^737^ in human airway epithelial cells (Fig. 6). Compared with controls, LMTK2 knockdown or substitution by mutagenesis of the LMTK2 phosphorylation site in CFTR (CFTR-Ser737A) increased the steady-state plasma membrane abundance of CFTR and decreased its endocytosis (Figs. 2, 4, 8). Conversely, inactivation by mutagenesis of the LMTK2 kinase (KM-LMTK2Δ-FLAG) increased the steady-state plasma membrane abundance of CFTR and decreased CFTR endocytosis compared with the WT-LMTK2Δ-FLAG and endogenous LMTK2 (Fig. 7). Third, endogenous LMTK2 was sufficient to phosphorylate CFTR-Ser^737^ and facilitate CFTR endocytosis because when compared with vector control, expression of the LMTK2 fragment with intact kinase activity WT-LMTK2Δ-FLAG did not increase CFTR-Ser^737^ phosphorylation, or the steady-state plasma membrane abundance of CFTR and did not attenuate CFTR endocytosis (Figs. 6 and 7).

Studies have shown that LMTK2 interacts with multiple proteins, including myosin VI (reviewed in Ref. 48). We have previously demonstrated that myosin VI facilitates CFTR endocytosis (26); and others have shown that myosin VI and LMTK2 regulate endocytic trafficking in other cell model systems (42, 43). Using the truncated LMTK2 fragments without the myosin VI binding domain and the tail domain allowed us to demonstrate for the first time that the cell surface density and endocytosis of CFTR are regulated by phosphorylation mediated by the LMTK2 kinase. Future studies may determine how the protein-protein interactions mediated by other LMTK2 domains influence the cell surface density and endocytosis of CFTR.

The phosphorylation of CFTR-Ser^737^ by PKA and AMPK inhibits CFTR channel function (32, 33). We do not know whether LMTK2 phosphorylation of CFTR-Ser^737^ inhibit the CFTR channel function in addition to reducing the cell surface density of CFTR. Similarly, the effects of AMPK and PKA phosphorylation of CFTR-Ser^737^ on the cell surface CFTR abundance are unknown. Hence, it remains to be determined whether the phospho-dependent inhibitory effects on CFTR-Ser^737^ are differentially mediated by each of the three kinases. Our data indicate that LMTK2 plays physiologically relevant role in regulating the phospho-dependent inhibitory CFTR-Ser^737^ site in human airway epithelial cells because similar to mutagenesis of the Ser^737^ site, LMTK2 knockdown produced a 2-fold increase in the cell surface abundance of CFTR and a 3-fold decrease in CFTR endocytosis (Figs. 2*B versus* 8C and Figs. 4*B versus* 8*B*, respectively). When compared with the LMTK2 knockdown, the kinase deficient LMTK2 fragment produced lesser effect on the cell surface abundance and endocytosis of CFTR (Figs. 7*E versus* 2*B* and Figs. 7*D versus* 4*B*, respectively). Our interpretation is that endogenous LMTK2 diminished the effects of the kinase deficient fragment when compared with LMTK2 knockdown. Increased AMPK activity was proposed to facilitate phosphorylation of Ser^737^ and inhibition of CFTR in non-stimulated epithelia (34, 35). Similarly, increased activity of LMTK2 at the apical surface is expected to inhibit CFTR-mediated Cl^−^ secretion at least in part by attenuating the cell surface density of CFTR. The LMTK2 gene polymorphisms and the protein kinase cdk5/p35 modulate LMTK2 function (68, 69); although, their effects on CFTR are unknown. By contrast, cigarette smoke exposure and chronic obstructive pulmonary diseases (COPD) reduce CFTR function by poorly defined mechanisms (reviewed in Ref. 70). Interestingly, cigarette smoke inhalation modifies the LMTK2 gene (69). Thus, examining effects of cigarette smoke exposure on LMTK2 may increase our understanding how this environmental toxin reduces CFTR function.

LMTK2 knockdown increased CFTR mediated Cl^−^ secretion in cells expressing WT-CFTR and ΔF508-CFTR (Figs. 3 and 10). In cells expressing ΔF508-CFTR this effect was mediated by stabilizing the mature, fully glycosylated, plasma membrane associated CFTR band C rescued by corrector VX-809 (Figs. 9 and 10). The effect of VX-809 was small but similar to effects observed in previous studies (17, 61, 71). We observed an interesting correlation between the effects of LMTK2 knockdown on the biochemical and functional rescue where a 10% increase of the band C abundance translated into a 50% rise of the forskolin/IBMX-stimulated *I*sc (Figs. 9 and 10). Clinical studies have already shown that targeted monotherapy by the CFTR corrector in patients homozygous for the ΔF508 mutation is promising but still inadequate likely because of decreased function and reduced plasma membrane stability of the pharmacologically rescued ΔF508-CFTR (16, 17, 21, 72). Hence, regulating LMTK2 phosphorylation of ΔF508-CFTR may play a role together with the CFTR corrector and potentiator to maximally rescue ΔF508-CFTR function in CF patients. LMTK2 knockdown did not increase the steady-state abundance of the partially glycosylated ΔF508-CFTR band B and it did not rescue ΔF508-CFTR in the absence of VX-809 (Fig. 9*C*). These data emphasize that the effects of LMTK2 knockdown on CFTR were mediated via alterations of post-maturational trafficking of CFTR during endocytic uptake and not during the biosynthetic processing of CFTR.

Using the primary differentiated human bronchial epithelial cells and a human polarized airway epithelial cell model CFBE41o- we have shown that LMTK2 targets to the apical and basolateral plasma membrane. Moreover, we provide novel information that the kinase domain dynamically regulated the endocytic uptake of LMTK2 in human airway epithelial cells (Fig. 7, *C* and *F*).

In summary our data provide direct evidence that CFTR endocytosis is regulated by phosphorylation mediated by the LMTK2 kinase domain. Because rescued ΔF508-CFTR has decreased plasma membrane stability we propose that targeting LMTK2 phosphorylation of CFTR may offer a novel approach to investigate the stability defect and to design pharmacological approaches to stabilize rescued ΔF508-CFTR. Our findings may also point to a new direction in elucidating the mechanisms of decreased CFTR function in lung diseases resulting from cigarette smoke inhalation.

## Footnotes

3 The abbreviations used are:

CFTR: cystic fibrosis transmembrane conductance regulator
ABC: ATP binding cassette
AMPK: adenosine monophosphate-stimulated kinase
LMTK: lemur tyrosine kinase
HBE: human bronchial epithelial.
